# Socio-Economic Status, Time Spending, and Sleep Duration in Indian Children and Adolescents

**DOI:** 10.1007/s10826-016-0557-8

**Published:** 2016-09-21

**Authors:** Radhika Bapat, Mitch van Geel, Paul Vedder

**Affiliations:** Institute of Education and Child Studies, Leiden University, PO Box 9555, Leiden, 2300RB the Netherlands

**Keywords:** Sleep, Socio-economic status, India, Screen time, School, Academic, Physical activity, Time budgets, Adolescents

## Abstract

In this article physical activity, screen time, and academic work are studied as mediators between socio-economic status and sleep duration among school children in India. Participants were 268 school children aged 10–15 from Pune, India. They were sampled from private schools and impoverished public schools. We found that the highest socio-economic status children reported almost an hour and a half less sleep than their lowest socio-economic status counterparts. The lower socio-economic status children reported more physical activity and screen time, and the higher socio-economic status children reported spending more time on academic work. Although screen time was negatively related to sleep duration, academic work was the strongest mediator between socio-economic status and sleep duration. Physical activity was not a significant mediator. In India, academic work is a strong predictor of a lower sleep duration among children and adolescents.

## Introduction

Several studies have revealed a link between socio-economic status (SES), here defined as family wealth and material resources (Boyce et al. 2006), and the sleep duration of children and adolescents (El-Sheikh et al. 2013; Mezick et al. 2008; Patel et al. 2010). Though in general, studies reveal that children from a lower SES sleep shorter (e.g., DeSantis et al. 2013), it has been found that in Turkey children with a higher SES sleep shorter, likely due to better access to television and videogames, and heavier homework demands (Arman et al. 2011). Consequences of insufficient sleep on youth include poorer scores on cognitive tasks (Bub et al. 1996), depression (Zhou et al. 2014) and even attempted suicide (Liu and Buysse 2006). Though sleep and sleeping problems, and the negative consequences thereof, have previously been studied in India (Bharti et al. 2006; Fatnani et al. 2015; Shaikh et al. 2009), predictors of sleep duration among children in India are relatively understudied.

One potential mediator between SES and sleep is physical activity. Many Western studies suggest that youth get insufficient sleep (El-Sheikh and Buckhalt 2015; Loessl et al. 2008; Smaldone et al. 2007) and insufficiently engage in physical activity (Biddle and Goudas 1998). Several studies suggest that more active youth sleep more (e.g., Brand et al. 2010; Ekstedt et al. 2013; Foti et al. 2011; Stone et al. 2013) although a few conclude that greater physical activity compromises sleep duration (Olds et al. 2011; Pesonen et al. 2011). The explanation for a positive relationship between sleep and physical activity is that physical activity helps to tire children and consequently promotes more sleep. Lower SES youth in the western world have been known to be less physically active than their higher SES counterparts (Patel et al. 2010). In India, high SES children may be less physically active than low SES children because of parents’ efforts to safeguard children from menacing neighborhoods in conjunction with a shortage of safe and accessible open spaces and playgrounds. Furthermore, academic activities are given preference over sports and even when it comes to going to school, affluent children do not need to walk or cycle because they have good access to motorized transport (Gupta et al. 2012).

Screen time is another potential mediator between SES and sleep. Television (TV) viewing, the use of computers and/or internet use, electronic gaming, and mobile telephones increasingly dominate the leisure time of children and adolescents (Coyne et al. 2013; Gradisar et al. 2013). Time spent on these activities have all been found related to delayed sleep times and shorter total sleep durations (for a review see Cain and Gradisar 2010). The explanation for the relations between these different forms of screen time and shorter sleep has mostly been similar between studies; screen time may displace time available for sleep or interfere with bedtime, it may arouse children before bedtime, and it may suppress the sleep promoting hormone melatonin via exposure to screens with bright light (Cain and Gradisar 2010; Hale and Guan 2015). Indian studies demonstrate that school-going children spend a large amount of time watching TV (Arya 2004; Ravikiran et al. 2014; Ray and Jat 2010). Most lower SES children in India have access to TV; a slum house is small and lacks facilities, but is typically equipped with a TV, which is often on (Sambasivan et al. 2009), and sometimes used as a sleeping aid (Ravikiran et al. 2014). Higher SES parents, instead, may eliminate a TV from their house altogether to facilitate their children’s academic success (Verma et al. 2002).

Particularly in India, academic demands could be a mediator between SES and sleep. School schedules and academic workloads have been found negatively related to sleep in studies from the United States (Crowley et al. 2007) and Europe (Hofman 2007; Russo et al. 2007), as well as in Asian countries like China (Chen et al. 2014) and Japan (Ohida et al. 2004). In India, especially in private schools catering to the higher SES children, parent, and teacher expectations make for rigid study routines spanning long hours, during, before, and after school (Deb et al. 2015; Larson and Verma 1999; Verma and Sharma 2003). High SES parents may even encourage children to study in lieu of sleeping (Ellinger and Beckham 1997). Furthermore, stress associated with academic activities may make it more difficult for children to fall asleep (Astill et al. 2013). Substituting study time for sleep may be less common for the lower SES children attending public schools, because teachers’ expectations for them are low, they do not assign much homework, and are often indifferent toward its completion altogether (Bempechat et al. 2011).

The current study examines the relationship between SES and sleep duration among low and high SES youth in India, focusing on three potential mediators: physical activity, screen time, and academic work. We hypothesize that overall it is the higher SES youth that will sleep less than the lower SES youth. We hypothesize that screen time is a mediator between SES and sleep time, with a negative relation between SES and screen time and negative relations between screen time and sleep duration. We hypothesize that physical activity is a mediator between SES and sleep duration, with higher SES youth reporting less sleep and less physical activity, and with this activity positively related to sleep duration. We also hypothesize that time spent on academic work is a mediator between SES and sleep duration, so that higher SES youth report more time studying than lower SES, and that time spent studying is negatively related to sleep duration. We will analyze which of the three mediators is the strongest mediator, but because there is insufficient literature on the strength of mediating mechanisms, we refrain from formulating hypotheses about this comparison.

## Method

### Participants

Fifteen classes of 5th to 9th graders from the lower and upper SES from two public (eight classes) and four private (seven classes) schools were included in the study. One public and one private school were segregated by gender and the remaining schools had mixed-sex classes. All classes from public schools were semi-English (medium of instruction was alternated between Marathi and English) and all private schools had English as their primary medium of instruction. We obtained parental and children’s consent for 442 children. We excluded 16 children from the sample due to learning disabilities or psychopathology. In the higher SES, 42 children withdrew during the course of the study. The reason that was mostly cited was that the study was seen as too time-consuming or bothersome. Furthermore, we tried to collect data on 3 days for time spending patterns, and we chose to only include those students who provided data on SES, and time spending on at least one weekday and a weekend day, because children’s sleeping patterns may be different between weekend and weekdays (Gradisar et al. 2011). This led to a further exclusion of 116 students. We were not able to gather data explaining why these children were absent from school on days we collected data. Informal discussions with classmates and teachers suggested that child labor or household chores were important reasons for absenteeism. In total 268 adolescents (60.6 % of consenting sample) were included in our analyses.

The adolescents ranged in age from 11 to 17 years (*M*age = 13.85, SD = 1.27). The analyses include152 boys (56.7 %) and 116 girls (43.3 %). For descriptive purposes SES categories were created using the Family Affluence Scale (Currie et al. 1997) and included three levels; the highest SES category (*N* = 85, 27.8 % female, *M*age = 13.86, SD = 1.24), the middle SES category (*N* = 104, 39.4 % female; *M*age = 13.75, SD = 1.17) and the lowest SES category (*N* = 79, 62.4 % female; *M*age = 13.95, SD = 1.43) (See Boyce et al. 2006 for the procedure). A *χ* ^2^ test of independence was calculated comparing SES levels with being in a public or private school. A significant effect, *χ* ^2^ (2, *N* = 268) = 187.47, *p* = .001 was seen; The vast majority of the lowest SES students (*N* = 77, 97.5 %) attended public schools, of the middle SES group, the vast majority attended private schools (*N* = 92, 88.5 %), and in the highest SES group, all children attended private schools.

### Procedure

We invited schools for participation based on the SES of their student body. We followed a convenience sampling approach and found the schools in a Pune school directory. We aimed to achieve a sample that included both children with a high and low SES, and therefore we sampled both public and private schools. Public schools do not charge a fee and are entirely run by government funding. Private aided and unaided schools charge fees, are run by private management, with the former funded partly by government grants and the latter without any government funding (Kingdon 2007). Public schools suffered frequent power outages and facilities were generally in a dilapidated state; toilets were ill maintained and often had no water, garbage was strewn across corridors, there was no storage space in classrooms, and oftentimes blackboards were neglected and in derelict condition. In the private schools, the facilities were comparatively abundant and tidy (see also De et al. 2011). Our team visited schools and provided coordinating staff with information about the aims and design of the study. Those schools that consented were included in the study. Parents and teachers were informed about the study mostly during parent—teacher meetings and sometimes personally. Children were informed group wise in class. Prior to data collection parents and students gave consent for participation. Anonymity was warranted to the participating schools and children. Children were assured that they could end their participation at any moment without any consequences. During the actual data collection, on Day 1, participants were explained the procedure which was to follow to ensure that they understood the protocol.

### Measures

#### Time use Pattern

Prior to the actual study, a pilot study was conducted. The results indicated that many students from public schools were unable to read or write age-appropriately. We therefore decided to use interviews to gather data. By recording interviews we ensured uniformity in data administration between the literate and illiterate school-going children. To aid children and interviewers, actual clocks were used. The “minute hand” was physically manipulated during the child’s narrations of events to symbolize the exact minutes that elapsed between activities and to maintain precision. Youth activities were determined by their responses to the questions “What was the main thing you were doing?” in 10-min epochs spanning 24-h starting from midnight of the previous day. Though some small activities may not be reported, overall time use diaries have been found a valid method to study children’s time spending (Larson and Verma 1999). The audio recordings were then coded verbatim by a different set of trained research assistants proficient in all three languages used by the children (Marathi, Hindi, English) into 24-h sheets. If children completed only a weekday and a day in the weekend, we computed an average of sleep for these 2 days.

If children reported all 3 days we first averaged the weekdays, and then took the average of the combined weekdays and the day in the weekend. For sleep, we included sleep at night and daytime napping. For screen time we coded time spent watching television, but also time spent using the internet, a computer, a tablet, or a video game console. For physical activity we coded individual and team based sports, training for sports, exercise, but also physically active play such as hide-and-seek, tag, or just running around with friends. For academic work, we coded study-time in school, tuition, and homework. Tuition here means any supervised activity to complete homework or improve academic performance, such as visiting after-school classes, working with a private tutor, but also receiving instructions about academic subjects from older family members. To get an indication of the interrater reliability, 10 randomly selected children from private schools (all higher SES) and ten randomly selected children from public school (all lower SES) were double coded. The intra-class correlation coefficients were .91 for sleep, .86 for time spent in physically active pursuits, .90 for screen time and .93 for time spent in academic activities.

#### SES

SES was measured using the “Family Affluence Scale” (FAS), which consists of four items (Currie et al. 1997). It is an index of material affluence and uses a composite score of four items: “Do your parents have a car or van?” responded to with “no,” “yes, one,” or “yes, two, or more;” “Do you have your own bedroom?” responded to with “no,” or “yes;” “how many computers does your family own?” responded to with “zero,” “one,” “two,” or “three or more;” and “how many times did you travel away on holiday with your family?” responded to with “not at all,” “once,” “twice,” or “more than twice.” The FAS has been found a valid indicator of SES in several studies (Boyce et al. 2006; Lin 2011; Molcho et al. 2007) and has been used in previous time budget studies (Carson et al. 2011).

### Data Analyses

A risk in time spending research is that a day only has 24 h, and more time spent on one activity means less time left over for other activities. Even though the displacement of sleep by other activities is in itself an important question, we attempted to account for this in several ways. Firstly, by entering all mediators simultaneously in the model the effects of each variable was controlled for SES, as well as other time spending mediators, showing how each time spending mediator individually is related to sleep over and above the other mediators. Secondly, we conducted interviews on three separate days on children’s time spending, and included only those children that reported at least a weekday and a weekend day in the analyses. This creates more possible variations in time spending patterns and reduces dependency between variables. Children performed other activities over the course of two or three days not included in the present analyses such as eating, grooming, chores, and time spent socializing with friends. Thirdly, we asked children about both primary activities (what were you doing?) and secondary activities (were you doing anything else at that time?) for each 10-min epoch of the day, again increasing variance and reducing dependency on other time spending variables.

Mediation was tested using the bootstrapping approach described by Preacher and Hayes (2004; 2008). In this approach samples are taken from the original dataset with replacement, and in each sample that is taken from the original dataset estimates of the indirect effects are calculated. The distributions of these estimates then serve as nonparametric approximations of the sampling distributions of the indirect effects. An important benefit of this method over the often used Baron and Kenny (1986) method is that there are no assumptions about the distributions of the data. For the current analyses we used 5,000 bootstrap samples and a confidence interval of 95 %. The age and gender of the children were added in the analyses as covariates. If the 95 % confidence interval did not contain zero, it was concluded that the indirect effect is significant at *p* < .05 (Preacher and Hayes 2004; 2008). All three mediators were entered in the model simultaneously, so that relations between the mediators were adjusted for in the analyses, and so that it could be statistically tested which of the three mediators (physical activity, screen time, academic work), was most strongly related to sleep time.

## Results

The means and standard deviations for the variables in this study are included in Table 1. When the groups are divided into three SES categories as per the Family Affluence Scale, we find the higher SES group sleeps significantly less than the lowest SES Group [*F*(2, 265) = 12.282, *p* = .001, *η* ^2^ = .085], that there are no significant differences between the SES groups in terms of physical activity [*F*(2, 265) = .217, *p* = .805, *η* ^2^ = .002], but there are significant differences on screen time [*F*(2, 265) = 10.524, *p* = .001, *η* ^2^ = .074], and academic work [*F*(2, 265) = 17,819, *p* = .001, *η* ^2^ = .119], with the lowest SES group reporting the most screen time and the least time spent on academic work. The correlations between the variables are reported in Table 2. In these analyses as well as in following analyses, FAS scores for SES are treated as a continuous variable.

**Table 1:** Means and standard deviations (in minutes) for sleep and time spending activities according to SES levels

		*N*	*M*	SD
Sleep	LSES	85	597.24	138.19
	MSES	104	534.21	114.72
	HSES	79	504.34	117.55
	Total	268	545.41	128.54
Active	LSES	85	126.23	93.10
	MSES	104	127.79	97.91
	HSES	79	119.21	77.36
	Total	268	124.76	90.48
Screen time	LSES	85	178.58	103.85
	MSES	104	154.74	75.99
	HSES	79	119.65	62.63
	Total	268	151.96	85.46
Academic	LSES	85	245.76	128.33
	MSES	104	339.27	116.13
	HSES	79	355.02	145.59
	Total	268	314.25	137.13

**Table 2:** Correlations between the main study variables

	Sleep	Active	Screen time	Academic
SES	−.325^**^	−.022	−.287^**^	.374^**^
Sleep		.167^**^	−.002	−.376^**^
Active			−.037	−.236^**^
Screen time				−.327^**^

* *p* < .05; **p < .01

The outcomes of the analyses of the role of mediators are reported in Table 3. We found a negative relation between SES and Sleep, which suggests that children with a higher SES slept shorter than children with a lower SES [point estimate = −16.01, (95 % CI: −21.70–−10.32)]. The results of our analyses showed that this relation between SES and sleep time was significantly mediated by screen time and academic work, but not by physical activity. SES was found significantly related to physical activity and screen time, with less affluent children reporting more physical activity and screen time than more affluent children. SES was also significantly related to academic work, with higher SES children reporting more academic work than lower SES children. Physical activity was not significantly related to sleep time, but both screen time and academic work were significantly related to less time reported sleeping. The directions of the effects are reported in Table 3, and the formal tests of significance for the mediation effects are reported in Table 4.

**Table 3:** Bootstrapped point estimates and confidence intervals for the mediation analysis, with SES as independent variable, sleep time as outcome, and physical activity, screen time and academic work as mediators^a^

	Point estimate	SE	95 % CI lower limit	95 % CI upper limit
Active^b^	−4.08	2.11	−8.23	.07
Screen time^b^	−9.89	2.06	−13.95	−5.84
Academic^b^	21.08	3.15	14.87	27.29
Active^c^	−.09	.08	−.25	.06
Screen time^c^	−.28	.08	−.44	−.12
Academic^c^	−.27	.05	−.37	−.16

^a^ the analyses are adjusted for age and gender

^b^ Relations between SES and active time, screen time, and academic work

^c^ Relations between active time, screen time, academic work and sleep time

**Table 4:** Results from the mediation analysis with SES as mediated variable and sleep duration as outcome variable^a^

	Indirect effect^b^	SE	95 % CI lower limit	95 % CI upper limit
Active	−.37	.37	−.10	1.45
Screen time	2.80	1.01	1.15	5.19
Academic	−5.64	1.49	−9.11	−3.18
Total	−2.48	1.60	−5.95	.46

^a^ the analyses are adjusted for age and gender

^b^ the indirect effect is the product of the a and b pathways

Using the procedure described by Preacher and Hayes (2008) we tested whether the strength of the mediation effects differed significantly. We found that academic time was a stronger mediator than screen time [point estimate = 8.44, 95 % CI (5.14 to 12.96)], and physical activity [point estimate = −6.02, 95 % CI (−9.80 to−3.29)]. Screen time was found to be a stronger mediator than physical activity [point estimate = 2.42, 95 % CI (.82 to 5.73)].

## Discussion

The current manuscript focused on the relation between SES and sleep, and potential mediators in this relation. Though past studies suggest that children with a lower SES sleep less (El-Sheikh et al. 2013; Patel et al. 2010), based on our review of sleep and potential mediators in India (Deb et al. 2015; Kuriyan et al. 2007; Ravikiran et al. 2014; Verma and Sharma 2003; Verma et al. 2002), we hypothesized that in India higher SES children would sleep less than lower SES children. We furthermore hypothesized that physical activity, screen time, and academic work would be mediators between SES and sleep duration.

In line with our first hypothesis, we found that children from a higher SES in India sleep less than children from a lower SES, which is similar to results earlier found in Turkey (Arman et al. 2011). In fact, higher SES youth slept on average for eight and a half hours a day, almost an hour and a half less than the average of their lower SES counterparts. Though we found that lower SES youth were more physically active, the results of our bootstrapping analyses did not support our hypothesis that physical activity was a mediator between SES and sleep duration. We already stated that there have been inconsistent results regarding the relation between physical activity and sleep (Olds et al. 2011; Pesonen et al. 2011). The effects of SES on sleep may be moderated under a number of circumstances, which would require an analysis of conditions under which physical activity is related to sleep rather than simply analyzing the relation between physical activity and sleep.

Screen time was found to be a significant mediator in the relation between SES and sleep time, with lower SES children reporting more screen time, and screen time negatively related to time spent sleeping. Paradoxically, even though higher SES reported less screen time, they also reported less sleep than the lower SES children. Consistent with our hypothesis, academic work was found to be a significant mediator between SES and sleep duration. To an extent, these results are comparable to what has been previously suggested by Arman et al. (2011) for children in Turkey; youth with a higher SES sleep less and heavier study demands are one explanation for this. However, in India the higher academic activity explained better than in Turkey why higher SES children slept less than lower SES children. For India we found that academic work was a stronger mediator than both screen time and physical activity. This immediately explains the paradox that poorer children in India report more screen time yet sleep more than the higher SES youth; the sleep loss lower SES youth may experience as a result of screen time is likely overshadowed by the sleep loss higher SES children experience as a result of academic work.

Much of the waking time of higher SES youth was spent in academic work, which was, on an average including a weekend day, just below 6 h a day. A previous study comparing Western and Asian cultural contexts of sleep (Liu et al. 2005) reported a marked variation in bedtimes and waking up times of Chinese school-going children in comparison to their US counterparts. Rigid school schedules, increased homework demands and an emphasis on academic achievement were seen as the justification. A similar emphasis on academics is reflected in other Asian cultures including Japan, Korea, Taiwan, and China (Corno and Xu 2004; Harnisch 1994; Kwok 2004). It appears that children from the Higher and Middle SES in India face exhaustive academic demands (Verma et al. 2002). This is not surprising in India, where children have their self-worth intertwined with their academic performance, where fear of school failure is predominant and where success as well as round-the-clock studying are rewarded (Deb et al. 2015).

Some limitations of the current study must be noted. Screen time was treated as a compound variable in our study, consisting of television viewing, electronic games, and computer-use. We made this choice because most of the children’s screen time consisted of television viewing, with electronic games and computer-use being reported far less; coding each category of screen time separately would likely have resulted in too little variation in the data, and we believe that our choice to create a compound variable is justified, given that all separate forms of screen time have been found related to overall screen time, and similar explanations have been offered for all these relations (Cain and Gradisar 2010). Nonetheless, given the increasing amounts of time children spend on the internet and on electronic video games (Coyne et al. 2013; Gradisar et al. 2013), it is better to address the potential effects of these variables separately from television viewing. A further limitation is that no special questionnaire for sleep was administered and the study lacked information about daytime sleepiness, weekday and weekend differences, sleep quality, sleep chronotypes and specific bedtime routines (El-Sheikh and Buckhalt 2015). Our study did not use objective measures like actigraphy, was not multimodal and lacked multiple informants. Also, because of our desire to sample understudied public schools in India many children were not retained in our final sample. We were able to sample these children one school day, but they went absent on other school days; we believe that these children may have been working, or kept home to help with domestic chores. Though this limits the generalizability of our sample, we also believe that this problem cannot be prevented when including the poorest school going children in India in a sample.

The current study adds to a growing body of literature that suggests that in Asia study related stress and activities might be so large that they intervene with children’s sleep (Corno and Xu 2004; Harnisch 1994; Kwok 2004). Furthermore, in India it is particularly the higher SES children that may sleep less as a result of school demands. Paradoxically, disturbed or decreased sleep is known to contribute to academic underachievement even in intelligent children and is therefore counterproductive (Erath et al. 2015). The most significant implication of the current study is for Indians to refrain from encouraging children to study in lieu of sleeping.
